# Life with Pain Revalued—A Therapist-Led Support Group for Patients with Chronic Non-Cancer Pain: A Pilot Feasibility Study

**DOI:** 10.3390/jcm15124641

**Published:** 2026-06-15

**Authors:** Maciej Klimasiński, Piotr Krajewski, Daria Metelkina, Nicole Goldsztajn, Andrea Trondsdatter Haugland, Malwina Prus-Zielińska, Marcin Wnuk

**Affiliations:** 1 Department of Palliative Medicine, Poznan University of Medical Sciences, os. Rusa 55, 61-701 Poznań, Poland; 2 Anesthesiology and Intensive Care Unit, Józef Struś Municipal Hospital, ul. Szwajcarska 3, 61-285 Poznań, Poland; 3 Department of Medicine, Poznan University of Medical Sciences, ul. Fredry 10, 61-701 Poznań, Poland; 4 Poznań College of Communications and Management, University of Communications and Management in Poznań, pl Wolności 18, 61-739 Poznań, Poland; 5 Faculty of Psychology and Cognitive Sciences, Adam Mickiewicz University, A. Szamarzewskiego 89/AB, 60-568 Poznań, Poland

**Keywords:** chronic pain, quality of life, mental health, depression, anxiety, spiritual well-being

## Abstract

**Introduction**. Chronic non-cancer pain is highly prevalent and profoundly diminishes quality of life. While pharmacological and interventional treatments are central, its psychosocial and spiritual dimensions remain under-addressed. This pilot study assessed the feasibility of a therapist-led support group intervention for patients with chronic non-cancer pain and explored preliminary psychospiritual outcomes. **Methods**. A two-arm, non-randomized pilot feasibility study was conducted among 58 outpatients of a university pain management clinic in Poland. Feasibility was assessed through recruitment, retention, attendance, and safety, while preliminary psychological and spiritual outcomes were evaluated using validated self-report instruments. The intervention group (*n* = 29) participated in eight group sessions combining psychoeducation, mindfulness-based techniques, and supportive dialogue inspired by the Simonton Method. The control group (*n* = 29) received standard care. Participants completed the Numeric Rating Scale to measure pain intensity, the Satisfaction with Life Scale, the Positive and Negative Affect Schedule, the WHOQOL-BREF, the Spiritual Well-Being Scale, the Generalized Anxiety Disorder Scale, and the Patient Health Questionnaire-9. **Results**. The intervention was feasible in terms of physician workload; however, patients adherence varied significantly. At baseline, the control group showed a significantly higher positive affect and existential well-being than did the intervention group. In exploratory within-group analyses, participants in the intervention group showed improved positive affect and reduced anxiety (*p* < 0.05), whereas existential well-being showed a trend toward improvement (*p* < 0.06). However, the self-selection design limits causal inferences. Nevertheless, participants reported social connectedness, meaning-making, and enhanced vitality. **Discussion**. This pilot feasibility study provides preliminary evidence that a therapist-led support group intervention integrating psychoeducation, mindfulness, and supportive components is practicable within multidisciplinary pain management. Further research in a larger, randomized trial is needed to evaluate adherence and safety, as well as clinical effects, more rigorously.

## 1. Introduction

### 1.1. Background of Chronic Pain

Chronic pain is a multifaceted condition characterized by its persistence over time and its resistance to conventional treatment. It is a growing public health problem worldwide. In Poland, it affects an estimated 8.5 million individuals, approximately 27% of adults, placing the national prevalence well above European and global averages. Rates rise to nearly 50% among older adults, with women, particularly those in rural areas, disproportionately affected [1]. Regardless of the cause, some chronic-pain patients have exhausted diagnostic and therapeutic options in primary or specialist care. When patients continue to report high pain intensity, they are referred to pain management clinics, where pharmacological and interventional treatments are optimized [2]. However, even when physical management is optimized, important psychological, social, and existential dimensions of living with chronic pain may remain insufficiently addressed.

### 1.2. Chronic Pain and Quality of Life

In pain management clinics, patients’ quality of life (QoL) is routinely assessed and is often found to be profoundly diminished [3]. For patients with chronic non-cancer pain refractory to conventional treatment, improving QoL may be considered as a central therapeutic goal [4]. In order to conceptualize quality of life, in this study, we framed it in a biopsychosocial–spiritual well-being model [5]. This is because persistent physical suffering may contribute to emotional exhaustion, spiritual distress, and social isolation, thereby affecting close relationships, community involvement and social roles [6]. Previous research has explored the bidirectional relationship between chronic pain and psychosocial well-being, as well as spiritual well-being, examining how supportive approaches may help improve quality of life in chronic-pain patients [7,8]. In addition to therapies focused on pain control, “psychological and spiritual well-being” (Box 1) is included within the framework of multimodal pain management [4,7].

Box 1 **Psychospiritual well-being [5,6,7].**

“Psychospiritual” is used as an umbrella term to refer to the combined psychological and spiritual domains of well-being assessed in this pilot study. It is understood as a multidimensional concept, which comprises of subjective psychological components, mental health indicators, and spiritual well-being.

### 1.3. Existing Interventions Targeting the Psychological Dimension of Well-Being

Cognitive Behavioral Therapy (CBT), as well as Acceptance and Commitment Therapy (ACT), are effective non-pharmacological interventions in this area [9,10,11]. However, these interventions do not benefit all patients equally, and their accessibility may be limited by factors such as cost, time requirements, and therapist availability, prompting the search for alternative or complementary strategies [10,12,13]. Williams et al. [10] analyzed 75 studies regarding the effectiveness of psychological therapies in chronic-pain management and found that CBT produces only small benefits. However, Sánchez-Gutiérrez et al. [14] reported that psychoeducational interventions alone can significantly improve quality of life in patients with chronic pain. Similarly, Darnall et al. [9] compared a single-session education intervention with eight sessions of CBT in adults with chronic pain and found that it was noninferior at 3 months. Cano-García et al. [15] argued that effective psychological interventions should have multiple components. Other psychological approaches, including mindfulness, biofeedback, hypnosis, and emotional awareness and expression therapy, have also garnered varying degrees of evidence across multiple pain conditions [13]. Taken together, these findings suggest that supportive and multi-component interventions may merit further exploration, particularly when access to more intensive psychological treatment is limited.

### 1.4. Existing Interventions Targeting the Spiritual Dimensions of Well-Being

According to the Polish Association for Spiritual Care in Medicine (PTODM) [16], spirituality is a multidimensional domain that includes transcendence, religiousness, existential concerns, and value-related dimensions (including relational and moral aspects). However, interventions that enhance spiritual well-being in ways that are acceptable to patients, regardless of religious affiliation, remain difficult to define [17]. Social connection, such as the presence of supportive relationships, is well recognized as a key determinant of living well with chronic pain [18]. In related fields, including peer-support programs such as 12-step groups, benefits have been linked to shared purpose, mutual support, and opportunities for meaning-making within a group setting [19,20,21]. Rather than adopting any specific religious or ideological framework, such observations support exploring supportive group interventions that allow participants to engage with meaning-related themes in a flexible and patient-centered way.

### 1.5. Developing a New Intervention That Would Target Both the Psychological and Spiritual Dimensions of Well-Being

There are limited published models of improving both the psychological and spiritual dimensions of well-being in chronic-pain outpatients [22]. The above-mentioned insights can be used to design an intervention aimed at improving the quality of life of patients with chronic non-cancer pain refractory to conventional treatment that would be “psychospiritual” in nature. Rather than providing a definitive test of efficacy, the present study was designed as a pilot feasibility study to examine whether a therapist-led support group intervention could be implemented in an outpatient setting and to explore preliminary outcomes.

In theory, such groups could help patients develop new peer relationships, cultivate compassion through shared discussion, exchange life stories, find meaning in their experiences, and foster hope and motivation [23]. The therapeutic process in the group intervention leverages group dynamics and participants’ interactions; therapists facilitate discussions by guiding the process, managing group dynamics, and maintaining the therapeutic focus. Interpersonal interactions and group processes are potential tools for insight, support, and change [24]. The distinctive feature of our proposed intervention is the intentional combination of structured group processes (social connection and mutual support) with a specific focus on spiritual well-being (while remaining non-doctrinal) implemented in a feasible, pragmatic, low-threshold outpatient format. This intervention uniquely applies this approach to chronic non-cancer pain of various etiologies and can be available to a diverse patient population. It also differs from generic peer support, which often lacks structured, theory-linked components and professional facilitation.

### 1.6. Aims of This Study

In contrast to standard CBT/ACT protocols, the present intervention was not primarily designed to improve pain coping per se. Importantly, the intervention was not intended as a pain-intensity reduction protocol; rather, it aimed to improve how patients function psychologically and spiritually while pain persists. To clearly identify outcomes, we further specify a measurable feature of each component of psychospiritual well-being. For subjective psychological components, these are cognitive (life satisfaction) and emotional (affect) measures [25]; for mental health, these are depression and anxiety indicators [26]; and for spiritual well-being, these are existential and religious measures [27]. However, to avoid inter-denominational and doctrinal inconsistencies, we purposefully excluded any religious features from the intervention.

Accordingly, this pilot study had two aims. First, it sought to examine the feasibility of a therapist-led support group intervention delivered in an outpatient setting. Second, it sought to explore the preliminary changes in psychospiritual outcomes, with existential well-being being the most primary expected outcome.

## 2. Methods

### 2.1. Participants and Setting

This study employed a two-arm, parallel, non-randomized pilot feasibility design to examine whether a therapist-led support group intervention could be delivered within routine outpatient pain management care. All patients in this clinic undergo specialized, pharmacological pain therapy. This involves personalized opioid titration. For patients presenting with a neuropathic pain component, antiepileptic (anticonvulsant) and antidepressant medications are utilized. If these standard treatments prove insufficient, medical cannabis or lidocaine infusions are prescribed. In addition to pharmacological care, all patients are systematically referred to physiotherapy. All participants are regularly screened and evaluated for depression and anxiety. If symptoms are identified, patients are promptly referred to a psychiatrist and psychotherapy.

The study protocol was submitted for review of the Bioethics Committee in February 2025. Between February and April 2025, patients receiving routine care at the Pain Management Clinic of the University Clinical Hospital in Poznań, Poland, were preliminarily assessed for their interest in participation in a support group; those who declined were considered for the control group. The study protocol was approved by the Bioethics Committee of the Poznań University of Medical Sciences (approval no. 212/25, dated 8 May 2025). Formal recruitment and data collection commenced after receiving ethical approval. Eligible individuals were invited verbally by the treating clinic physician (the first author). No external recruitment (e.g., flyers or specialist referrals) was used. Group allocation was based on self-selection: patients who volunteered for the intervention participated in the group sessions, whereas those in the control group continued usual care without group therapy. It should be noted here that a non-randomized self-selection design introduces important selection bias.

Inclusion criteria were as follows: age ≥ 18 years; non-cancer pain persisting for ≥3 months despite conventional treatment, regardless of etiology, diagnosed by a pain specialist; and pain intensity > 4 on the Numeric Rating Scale (NRS), on average, over the previous month. Psychiatric comorbidity was screened via the tools described below; no formal DSM-based psychiatric diagnoses were collected in this pilot study. There were no additional exclusion criteria. Participants were categorized into three age categories: 20–40 years, 40–65 years, and ≥65 years. Baseline measures, described below, were collected in both groups prior to the initiation of the intervention. All participants received standardized written information about the study and provided written informed consent prior to enrollment. They were also informed that they could discontinue attending the group at any time, without giving any reason.

### 2.2. Intervention

The intervention consisted of eight 2 h group sessions held approximately every two to three weeks. The control group received continuation of standard pain clinic care; no additional psychological interventions, group therapy, or pastoral care referrals were initiated in this arm during the study period.

The intervention was guided by a brief, structured manual developed for this pilot (Box 2), informed by a Simonton-inspired approach. Originally developed for cancer patients, the Simonton Method is a psycho–oncological approach rooted in CBT. It aims to identify and restructure unhealthy, despair-inducing beliefs about illness, utilize relaxation and guided imagery to reduce physical and emotional tension, and help patients discover a sense of purpose and meaning despite their medical limitations. Although chronic non-cancer pain is not terminal, these patients face a severely diminished quality of life and daily struggles that closely mirror those of oncological patients (e.g., severe physical suffering, functional limitations, and learned helplessness). Furthermore, chronic pain is heavily modulated by psychological distress and catastrophizing, which act as central pain sensitizers. The Simonton Method directly addresses these mechanisms by shifting the focus from a medical “cure” to long-term coping, thereby helping patients downregulate pain perception and improve their emotional resilience [28].

Box 2 **A brief, structured manual developed for this pilot program.**

**Psychoeducational components:**

- Gratitude practice—reflection on uplifting or positive events to strengthen adaptive emotional states.
- Engagement in vital activities—identifying and practicing personally meaningful activities (e.g., walking in nature, meaningful conversations, gardening, listening to music, creating art, cycling, or enjoying a preferred beverage) to foster joy and mental calm.
- Development of emotional awareness—recognizing emotions as indicators of unmet needs and antecedent thoughts.

**Mindfulness/imagery practices**

- Mindful breathing with guided imagery—adapted from Simonton’s techniques.

**Supportive components:**

- Recognition of the impact of emotional distress on engagement in life-enhancing activities, joy, tranquility, and fulfillment.
- Identification and reframing of maladaptive beliefs that reduce motivation for active engagement.
- Exploration of personal sources of hope, strategies to cultivate and sustain it, and approaches to protect optimism from persistently discouraging influences.

Importantly, this intervention was not intended as a direct replication of the original Simonton Method program. Rather, it used selected techniques and overarching principles adapted to the context of a public outpatient pain clinic. This format was used to promote consistency of delivery across sessions and facilitators while allowing flexibility to respond to group dynamics. The components of the manual were not delivered as isolated modules assigned to specific sessions. Instead, they were integrated across all eight meetings within a repeated session structure. Each session typically included: (1) a brief check-in and reflection on recent experiences; (2) one or two experiential or psychoeducational elements; (3) facilitated group dialogue; and (4) a closing summary. The intervention was designed on the assumption that any potential benefits could arise both from specific components and nonspecific group processes (universality, instillation of hope, and group cohesion).

Sessions were facilitated by two licensed psychologists and one licensed occupational therapist. The psychologists had prior training in group therapy and the Simonton approach. The occupational therapist contributed mainly an activity-oriented perspective. Given its pragmatic, clinic-based format, the program is best described as a structured support group facilitated by therapists, rather than as a fully manualized psychotherapy protocol. Intervention fidelity was monitored throughout the program by the study coordinator (MWK). Fidelity checks were conducted regularly during the intervention period to ensure that planned components were delivered and that the session structure was maintained across meetings. There was no validated checklist, but any deviations or necessary adaptations were discussed with facilitators to maintain adherence to the manual while preserving responsiveness to participants’ needs.

### 2.3. Concomitant Treatment Changes

To minimize potential confounding, participants continued their usual pain management throughout the study. No significant changes in pharmacological therapy or interventional procedures were introduced in either study arm during the intervention period. Treatment stability was verified by the first author, who was the sole consulting physician during the study period and who did not modify pharmacological or interventional pain management in either study arm. This approach was intended to reduce the likelihood that any observed between-group or within-group differences were related to concurrent changes in medical management. As no treatment modifications occurred, concomitant care was not included as a covariate in the statistical analyses.

### 2.4. Measures

Study outcomes were operationalized using the following validated self-report measures administered in Polish:

- Pain intensity—Evaluated with two Numeric Rating Scale (NRS) items: current pain and on-average pain over the past month (0 = no pain to 10 = worst possible pain).
- Life satisfaction—Measured with the Polish adaptation [29] of the Satisfaction with Life Scale (SWLS) [30], a unidimensional tool comprising five items rated on a 7-point Likert scale (1 = strongly disagree to 7 = strongly agree; total score 5–35; higher scores indicate greater life satisfaction).
- Positive and negative affect—Assessed using the Polish short form [31] of the Positive and Negative Affect Schedule (PANAS) [32]. The short form contains the five strongest loading items for positive affect and negative affect from the 20-item version of this measure [33]. Within PANAS, the responses are given on a 5-point Likert scale (from 1 = a little or none to 5 = very frequently).
- Quality of life—Measured with a single global quality-of-life item from the WHOQOL-BREF [34], rated on a 5-point Likert scale (1 = very low to 5 = very high).
- Spiritual well-being—Evaluated using the Polish version [35] of the Spiritual Well-Being Scale (SWBS) [27], which has two subscales: Religious Well-Being (6 items) and Existential Well-Being (5 items). Each item is answered on a 6-point Likert scale (from 1 = strongly disagree to 6 = strongly agree).
- Anxiety—Measured with the Polish adaptation [36] of the Generalized Anxiety Disorder Scale (GAD-7) [37], a unidimensional 7-item tool with responses on a 4-point Likert scale (0 = not at all to 3 = nearly every day). Higher scores indicate more severe anxiety symptoms.
- Depression—Assessed using the Polish version [38] of the Patient Health Questionnaire-9 (PHQ-9), a unidimensional measure with nine items scored from 0 (not at all) to 3 (nearly every day) over the past two weeks. Higher scores reflect greater depressive symptom severity.

All measures were administered at baseline (Time 1) and after the final group therapy session (Time 2) in both the intervention and control groups. In addition to quantitative measures, participants in the intervention were invited to share brief, informal reflections regarding their experience.

### 2.5. Statistical Analyses

As this was a pilot feasibility study, the analyses were primarily descriptive and exploratory. Outcomes were not formally prespecified as primary and secondary at study initiation. However, existential well-being was treated as the conceptually central outcome because it most closely reflected the intervention’s meaning-oriented focus. Between-group differences were examined at baseline (Time 1) and post-intervention (Time 2), and within-group changes from Time 1 to Time 2 were also assessed. Given the modest sample size and the non-normal distribution of several variables, as assessed using the Shapiro–Wilk test [39], non-parametric methods were used throughout. Between-group comparisons were performed using the Mann–Whitney U test, and within-group changes were examined using the Wilcoxon signed-rank test [40].

### 2.6. AI Statement

During the preparation of this manuscript, the first author used GPT-5.4 for the purpose of minor linguistic refinements, including spell-checking and grammatical corrections. The authors have reviewed and edited the output and take full responsibility for the content of this publication.

## 3. Results

A total of 63 patients were enrolled in the study, including 29 participants in the control group and 34 volunteers for the intervention group. As planned, the whole intervention was conducted over 4 months. During the study period, four participants withdrew from the intervention (reasons not recorded), and one participant died. Therefore, a total of 58 patients were included in the final analysis. Sociodemographic characteristics and diagnoses are detailed in Table 1. Osteoarthritis and intervertebral disc disease were most prevalent in both the intervention and control arms. The control group consisted of 29 individuals, including 16 women (55.2%), 12 men (41.4%), and one transgender man (3.4%). The mean age in the control group was 56.7 years (range: 18–84 years). Among the participants of the intervention arm, there were 21 women (72.4%) and 8 men (27.6%) who completed the course of group therapy meetings. The mean age was 57.1 years (range: 23–87 years). Descriptive statistics for the study participants are presented in Table 2. Routine safety monitoring was performed throughout the intervention period. During each session, the facilitating therapist monitored participants for signs of increased distress. However, no referrals to individual psychotherapy were made during the study period. Attendance averaged 4.8 sessions (SD = 1.7); therefore, the actual group size per session varied.

The intervention was feasible in terms of physician workload. The clinic physician’s role was limited to providing the coordinator with the telephone numbers of the patients who had volunteered to participate. The coordinator then contacted them with further details. The coordinator was an author of this paper and therefore, did not receive payment. The licensed psychologists received payment comparable to a standard psychotherapy fee. The licensed occupational therapist was also an author of this study and thus, did not receive payment. An appropriate room had to be rented, which constituted the last additional cost.

To verify differences between the two groups, as well as potential improvement in well-being in the experimental group caused by the intervention and the lack of change in this outcome in the control group, the non-parametric Mann–Whitney test and a Wilcoxon signed-rank test were used. The Shapiro–Wilk test was designed to determine whether a variable was normally distributed [39]. The significant results for the Shapiro–Wilk test for subjective quality of life and religious well-being in both groups (see Table 3 and Table 4) and additionally, for positive affect in the experimental group (see Table 3), were the premise to use a non-parametric Mann–Whitney test and a Wilcoxon signed-rank test [40].

At baseline (Time 1), the intervention and control groups did not differ significantly in regards to most study variables; however, significant between-group differences were observed for positive affect and existential well-being (see Table 5). In both cases, participants in the control group had higher baseline scores than those in the intervention group. Within the intervention group, comparisons between Time 1 and Time 2 showed statistically significant increases in positive affect and statistically significant reductions in anxiety (*p* < 0.05; see Table 6). Existential well-being also increased, but this change did not reach conventional statistical significance (*p* = 0.056). In the control group (see Table 6), no statistically significant differences were observed between Time 1 and Time 2. At Time 2, after the intervention period, only one statistically significant between-group difference was found, namely for positive affect. Although positive affect increased within the intervention group, participants in this group continued to score significantly lower on positive affect than those in the control group at Time 2, as was also the case at baseline (see Table 5 and Table 6). No statistically significant between-group differences were observed for anxiety or existential well-being at Time 2, despite the within-group reduction in anxiety and increase in existential well-being observed in the intervention group (see Table 6).

Some brief anecdotal quotations were recorded by facilitators during sessions to provide a descriptive context for participant experiences. They were not subjected to systematic qualitative frameworks such as coding, thematic analysis, or saturation procedures, and are included solely as illustrative material rather than as formal qualitative findings. Several participants described the group as an opportunity for meaningful social contact. For example, one noted, “I started to notice the good things around me.” Another reflected, “I met people who can listen to what I have to say.” Others emphasized the value of social support: “I have new friends now. The previous ones turned away from me.” Some reported increased confidence: “I am gaining the courage to go out. Until now, I was ashamed of my illness; the group gave me confidence.” Participants also described feeling accepted, as illustrated by the statement, “I felt understood here; I really appreciate that no one judged me.”

## 4. Discussion

### 4.1. Strengths

An important finding of the study is the practical feasibility and implementation of the intervention. It was successfully delivered in a real-world outpatient pain clinic, showing that such a program is practical, and patients are willing to participate. The group was facilitated by licensed psychologists and an occupational therapist, ensuring professional guidance.

Although our study was not designed to provide definitive evidence of efficacy, the findings offer preliminary support for the view that participation in such a group may be associated with positive outcomes in selected aspects of psychospiritual well-being. Participants in the intervention group experienced improvements in positive affect and reductions in anxiety. There was a trend toward improved existential well-being, and participants reported enhanced social connectedness, meaning-making, and vitality.

The intervention structure is another asset of the study. The sessions combined psychoeducation, mindfulness, and supportive dialogue, inspired by the Simonton Method, and were open to patients regardless of religious background. The group format fostered social support, hope, and motivation through shared experiences and group dynamics. The observed findings may be understood in terms of both specific and non-specific therapeutic mechanisms, as described by Yalom [41]. In the context of chronic pain, shared narratives and mutual validation may support meaning-making processes by helping participants reinterpret suffering, clarify personal values, and re-engage with meaningful aspects of life. Social connectedness itself may have played an important role, as the group format appeared to offer participants a sense of being heard, understood, and less alone in their experience of pain.

### 4.2. Areas for Improvement

First, this pilot study used a self-selection design. The intervention arm included patients who volunteered to participate in the support group, whereas the control arm consisted primarily of patients who declined participation. This allocation procedure introduces a risk of selection bias and limits internal validity because those who chose not to enroll may have differed systematically in terms of motivation, expectations, symptom burden, receptivity to psychosocial support, or baseline psychological functioning from those who did. In this particular case, the control group showed higher existential well-being and positive affect at Time 1. Baseline differences could have influenced the results, inflating or masking the true effect of the intervention. It is also possible that the lower level of positive affect and existential well-being in the experimental group could have been an “opened window” for positive change. Similarly, the lack of randomization limits the internal validity of the study and diminishes the ability to make causal inferences. A better design would be to compare an experimental group of volunteers to another group of volunteers randomized to a waiting list. However, this would not solve another limitation regarding our control group, i.e., it did not receive matched attention; therefore, some observed changes may reflect the abovementioned non-specific therapeutic factors, such as expectancy effects and interpersonal support, rather than the specific content of the intervention itself.

Second, validity is limited because our sample size was small, and the study was not powered to detect all possible effects. Additionally, multiple outcomes, two timepoints, and both between-group and within-group comparisons increase the risk of Type I error and limit inferential confidence, especially in the absence of correction for multiple testing.

Third, the intervention was only partially manualized, and there was no validated checklist to ensure fidelity. Also, generalizability is limited by the single-center setting and the specific cultural context in which the study was conducted. Although these features reflect the pragmatic, real-world outpatient format of the study, they reduce replicability.

Fourth, attendance varied across sessions, with a mean of 4.8 out of 8 sessions, which likely reflects the substantial symptom burden typical of patients with chronic pain. An additional practical factor may also have influenced attendance: the sessions were held in a room located at a distance from the main clinic building, which was an additional logistical barrier for some participants. For patients with chronic pain, even relatively short travel outside essential medical appointments may be difficult, particularly when mobility is limited or when attendance depends on assistance from relatives or other caregivers. Such support may not always have been available, which could have contributed both to variability in attendance and to difficulties in completing the program. As stated previously, retention was incomplete: four participants withdrew from the intervention, and one participant died during the study period. Given that this indicator was low (approximately 5%) [42], it should not have significantly affected the results, even in a relatively small sample. Nevertheless, the exact reasons for withdrawal and session non-attendance were not systematically recorded. This underscores the need for future full-scale studies to document discontinuation reasons and attendance patterns more systematically.

Finally, validity is also limited because of a short follow-up. The study did not assess the long-term effects or sustainability of the intervention’s benefits. Moreover, no significant effects were observed for life satisfaction, depression, or several other exploratory outcomes, suggesting that brief group-based psychospiritual support may have a more immediate influence on affective and existential functioning than on broader or more persistent dimensions of well-being. The differential pattern observed for existential and religious well-being deserves specific consideration. In line with the PTODM framework, spirituality may be understood as a broad and multidimensional domain that includes religiousness, existential concerns, and value-based dimensions, including relational and moral aspects. Existential well-being may be understood as one measurable expression of adaptive meaning-making. Regardless of whether a person holds a religious or non-religious worldview, supportive group participation may facilitate a process of reinterpreting suffering, clarifying life priorities, and reconnecting with valued aspects of life [20,21]. A similar mechanism may have operated in the present intervention: listening to others’ life stories and being heard in return may have helped some participants reframe persistent pain within a broader sense of meaning and personal value. From this perspective, future research on psychospiritual interventions for chronic pain may benefit from maintaining a non-doctrinal, meaning-oriented focus that remains open and acceptable to patients with diverse worldviews.

Despite these limitations, this pilot study offers promising evidence for the feasibility and potential benefits of a therapist-led support group intervention in chronic pain care and provides a basis for future research. This intervention should be evaluated in a larger, preregistered randomized controlled trial including standardized procedures, facilitator supervision, attention control, follow-up assessment, more systematic recording of withdrawal reasons, attendance monitoring, and other feasibility indicators. A future non-pilot study should be conducted using a larger sample, including correction for multiple testing, to strengthen inferential confidence. Such refinements would allow a more rigorous assessment of both implementation and potential clinical effects over time.

## 5. Conclusions

This non-randomized pilot feasibility study suggests that a therapist-led, group-based intervention can be implemented in an outpatient chronic-pain setting, possibly offering a practicable way of addressing the psychospiritual aspects of functioning, alongside the physical symptoms. Conducted in a publicly funded pain clinic in Poland, this pilot study provides preliminary evidence that a structured support group integrating psychoeducational, experiential, and meaning-oriented elements may be associated with some beneficial changes in selected psychospiritual outcomes in patients with chronic non-cancer pain. Participation was associated with no significant improvement in life satisfaction, depressive symptoms, negative affect, or religious well-being. Even though we recorded increased positive affect, reduced anxiety, and a tentative improvement in existential well-being, given the pilot design, self-selection, non-randomized allocation, and lack of an attention-matched control condition, these findings should be regarded as preliminary. A larger randomized study is needed to evaluate this intervention more rigorously, clarify its mechanisms, and determine whether its effects are sustained over time.

## Figures and Tables

**Table 1:** Sociodemographic data and diagnoses of study arms.

Section/Variable	Study Arm N (%) or Mean ± SD	Control Arm N (%) or Mean ± SD	All N (%) or Mean ± SD
General characteristics			
N of participants	29	29	58
Age (years)	57.1 ± 18.3 (range: 23–87)	56.7± 19.5 (range 18–84)	57.1 ± 18.7
Women	21 (72.4%)	16 (55.2%)	37 (63.8%)
Household size	1.9 ± 1.1	2.3 ± 1.2	2.1 ± 1.1
Education			
Primary	2 (6.9%)	4 (13.8%)	6 (10.3%)
Secondary	15 (51.7%)	12 (41.4%)	27 (46.6%)
Tertiary	5 (17.2%)	8 (27.6%)	13 (22.4%)
Vocational	7 (24.2%)	5 (17.2%)	12 (20.7%)
Employment status			
Employed	6 (20.7%)	7 (24.1%)	13 (22.4%)
Retired	16 (55.2%)	19 (65.5%)	35 (60.3%)
Student	4 (13.8%)	3 (10.3%)	7 (10.4%)
Unemployed	3 (10.3%)	1 (3.4%)	4 (6.9%)
Marital status			
Partnered/Married	11 (37.9%)	16 (55.3%)	27 (46.6%)
Single	6 (20.7%)	3 (10.3%)	9 (15.6%)
Widowed	6 (20.7%)	3 (10.3%)	9 (15.6%)
Divorced	6 (20.7%)	7 (24.1%)	13 (22.2%)
Diagnoses			
Osteoarthritis (knee/hip/spine)	12 (41%)	13 (45%)	25 (43%)
Intervertebral disc disease	9 (31%)	8 (28%)	17 (29%)
Fibromyalgia	3 (10%)	3 (10%)	6 (10%)
Peripheral neuropathy	2 (7%)	2 (7%)	4 (7%)
Post-traumatic/post-surgical	2 (7%)	2 (7%)	4 (7%)
Headache/migraine	1 (4%)	1 (3%)	2 (3%)
Pain intensity on Numeric Rating Scale (NRS)			
NRS—current	5.8 ± 2.0	5.3 ± 2.0	5.6 ± 2.0
NRS—on average over the past month	6.6 ± 2.0	5.4 ± 1.6	6.0 ± 1.9
Religious affiliation			
Catholic	23 (79%)	24 (83%)	47 (81%)
Other	1 (4%)	0 (0%)	1 (2%)
None	5 (17%)	5 (17%)	10 (17%)

**Table 2:** Descriptive statistics for representatives of the experimental group and the control group at Time 1 (*n* = 58).

Variable	Minimum	Maximum	Mean	Standard Deviation	Skewness	Kurtosis	Alpha Cronbach Coefficient
Current pain intensity	2	9	5.52	2.04	0.13	−1.13	-
Satisfaction with life	5	24	14.96	4.64	−0.24	−0.04	0.84
Positive affect	5	25	14.41	4.66	0.25	−0.41	0.75
Negative affect	6	25	16.38	5.24	−0.15	−1.03	0.88
Subjective quality of life	1	5	2.95	1.08	−0.41	−0.61	-
Religious well-being	5	36	19.61	10	0.02	−1.16	0.97
Existential well-being	6	35	19.93	7.42	−0.01	−0.78	0.87
Anxiety	7	35	23.41	6.98	−0.36	−0.53	0.93
Depression	0	26	14.4	5.97	−0.24	−0.36	0.85

**Table 3:** The results of the Shapiro–Wilk test of normal distribution of the variables in the experimental group (*n* = 29).

Variable	Shapiro–Wilk Statistics	*p* Value
Satisfaction with life	0.98	0.82
Positive affect	0.92	0.03
Negative affect	0.95	0.16
Subjective quality of life	0.91	0.02
Religious well-being	0.90	0.01
Existential well-being	0.97	0.55
Anxiety	0.96	0.41
Depression	0.95	0.25
Current pain intensity	0.91	0.01

**Table 4:** The results of the Shapiro–Wilk test of normal distribution of the variables in the control group (*n* = 29).

Variable	Shapiro–Wilk Statistics	*p* Value
Satisfaction with life	0.96	0.28
Positive affect	0.98	0.72
Negative affect	0.94	0.11
Subjective quality of life	0.85	<0.01
Religious well-being	0.89	<0.01
Existential well-being	0.96	0.27
Anxiety	0.97	0.52
Depression	0.97	0.59
Current pain intensity	0.93	0.07

**Table 5:** Comparison of the results of the Mann–Whitney statistic between the experimental (*n* = 29) and control group (*n* = 29) at Time 1 and Time 2.

Variable	Mean and SD Control Group	Mean and SD in Experimental Group	Mann–Whitney Test	*p*
Results regarding baseline (time 1)				
Satisfaction with life	15.44 (4.36)	14.48 (4.93)	0.67	0.50
Positive affect	16.62 (4.03)	12.20 (4.22)	3.93	0.00
Negative affect	15.48 (5.13)	17.27 (5.27)	−1.39	0.16
Subjective quality of life	3.17 (1.03)	2.72 (1.09)	1.69	0.09
Religious well-being	18.24 (8.85)	21.03 (11.04)	−1.05	0.29
Existential well-being	22.31 (7.79)	17.72 (6.43)	2.29	0.02
Anxiety	23.24 (7.22)	23.58 (6.84)	−0.17	0.86
Depression	13.79 (6.31)	15.00 (5.65)	−1.06	0.28
Current pain intensity	5.86 (1.10)	5.75 (2.06)	−0.83	0.40
Results regarding baseline (time 2)				
Satisfaction with life	15.62 (4.25)	14.44 (6.05)	1.41	0.15
Positive affect	16.93 (3.43)	14.06 (5.13)	2.32	0.02
Negative affect	15,41 (4.91)	16.27 (5.02)	−0.79	0.42
Subjective quality of life	3.10 (0.93)	2.86 (1.27)	0.31	0.75
Religious well-being	17.82 (8.58)	21.27 (11.93)	−1.03	0.30
Existential well-being	21.86 (7.82)	19.24 (6.36)	1.53	0.12
Anxiety	21.41 (7.36)	21.27 (8.69)	0.60	0.54
Depression	13.20 (5.74)	15.24 (6.19)	−1.16	0.24
Current pain intensity	5.68 (2.10)	5.82 (2.31)	−1.21	0.22

Note: SD = standard deviation.

**Table 6:** The results of the Wilcoxon test pre- and post-intervention within the experimental group (*n* = 29) and control group (*n* = 29).

Variable	Mean and SD Pre-Intervention	Mean and SD Post-Intervention	Wilcoxon Test	*p*
Results in experimental group				
Satisfaction with life	14.48 (4.93)	14.24 (5.89)	−0.56	0.57
Positive affect	12.20 (4.22)	14.41 (4.96)	3.11	0.00
Negative affect	17.27 (5.27)	16.27 (5.02)	−1.03	0.30
Subjective quality of life	2.72 (1.09)	2.96 (1.29)	1.70	0.08
Religious well-being	21.03 (11.04)	20.71 (11.82)	−0.60	0.54
Existential well-being	17.72 (6.43)	19.55 (5.81)	1.91	0.06
Anxiety	23.58 (6.84)	20.06 (8.50)	−2.68	0.00
Depression	15.00 (5.65)	15.17 (6.08)	0.16	0.86
Current pain intensity	5.75 (2.06)	5.75 (2.19)	−0.08	0.82
Results in control group				
Satisfaction with life	15.44 (4.36)	15.62 (4.25)	0.13	0.89
Positive affect	16.62 (4.03)	16.93 (3.43)	0.62	0.53
Negative affect	15.48 (5.13)	15.41 (4.91)	0.12	0.89
Subjective quality of life	3.17 (1.03)	3.10 (0.93)	−0.50	0.61
Religious well-being	18.24 (8.85)	17.82 (8.58)	−0.69	0.48
Existential well-being	22.31 (7.79)	21.86 (7.82)	−0.57	0.56
Anxiety	23.24 (7.22)	21.41 (7.36)	−1.10	0.26
Depression	13.79 (6.31)	13.20 (5.74)	−0.70	0.48
Current pain intensity	5.27 (2.03)	5 (2.29)	−0.32	0.74

Note: SD = standard deviation.

## Data Availability

The original contributions presented in this study are included in the article. Further inquiries can be directed to the corresponding author.
